# The sequence of the repetitive motif influences the frequency of multistep mutations in Short Tandem Repeats

**DOI:** 10.1038/s41598-023-32137-y

**Published:** 2023-06-24

**Authors:** Sofia Antão-Sousa, Nádia Pinto, Pablo Rende, António Amorim, Leonor Gusmão

**Affiliations:** 1 Instituto de Investigação e Inovação em Saúde (i3S), University of Porto, Porto, Portugal; 2 Institute of Molecular Pathology and Immunology of the University of Porto (IPATIMUP), Porto, Portugal; 3 Department of Biology, Faculty of Sciences of University of Porto (FCUP), Porto, Portugal; 4 DNA Diagnostic Laboratory (LDD), State University of Rio de Janeiro (UERJ), Rio de Janeiro, Brazil; 5 Center of Mathematics of University of Porto (CMUP), Porto, Portugal

**Keywords:** Mutation, Population genetics

## Abstract

Microsatellites, or Short Tandem Repeats (STRs), are subject to frequent length mutations that involve the loss or gain of an integer number of repeats. This work aimed to investigate the correlation between STRs’ specific repetitive motif composition and mutational dynamics, specifically the occurrence of single- or multistep mutations. Allelic transmission data, comprising 323,818 allele transfers and 1,297 mutations, were gathered for 35 Y-chromosomal STRs with simple structure. Six structure groups were established: ATT, CTT, TCTA/GATA, GAAA/CTTT, CTTTT, and AGAGAT, according to the repetitive motif present in the DNA leading strand of the markers. Results show that the occurrence of multistep mutations varies significantly among groups of markers defined by the repetitive motif. The group of markers with the highest frequency of multistep mutations was the one with repetitive motif CTTTT (25% of the detected mutations) and the lowest frequency corresponding to the group with repetitive motifs TCTA/GATA (0.93%). Statistically significant differences (α = 0.05) were found between groups with repetitive motifs with different lengths, as is the case of TCTA/GATA and ATT (p = 0.0168), CTT (p < 0.0001) and CTTTT (p < 0.0001), as well as between GAAA/CTTT and CTTTT (p = 0.0102). The same occurred between the two tetrameric groups GAAA/CTTT and TCTA/GATA (p < 0.0001) – the first showing 5.7 times more multistep mutations than the second. When considering the number of repeats of the mutated paternal alleles, statistically significant differences were found for alleles with 10 or 12 repeats, between GATA and ATT structure groups. These results, which demonstrate the heterogeneity of mutational dynamics across repeat motifs, have implications in the fields of population genetics, epidemiology, or phylogeography, and whenever STR mutation models are used in evolutionary studies in general.

## Introduction

Microsatellites, or short tandem repeats (STRs), consist of tandemly arrayed 1–6 base pairs (bp) motifs. These are among the most useful and commonly employed genetic markers in population, forensic, or conservation genetics^1^, due to their variability and ubiquity. Their instability has relevant medical implications, being linked to cancer^2^ and to many other diseases. Namely, there are over 40 neurological, neurodegenerative, and neuromuscular disorders determined by repeat expansions of STRs at coding and non-coding regions^3^.

STRs undergo rapid length changes due to the insertion or deletion of one or multiple repeat units^1,3^. The primary mutational mechanism thought to lead to changes in STR length is polymerase template slippage during DNA replication^4,5^. A distinct pathway is associated with unequal crossing over, which may happen due to strand mispairing during recombination^6^.

The stepwise mutation model (SMM) was introduced by Ohta and Kimura^7^ and Wehrhahn^8^, suggesting mutational dynamics of STRs where parental alleles gain or lose a single repeat when transmitted to the offspring. The possibility of multistep changes was also considered, although at a much lower rate. Indeed, some works showed that the proportion of multistep mutations represents 1% of the detected mutations for tri- and tetranucleotide STRs, increasing this figure to 30% for dinucleotide STRs^9,10^. The SMM has been used to model STR mutation and evolution and has been applied in diverse areas such as population genetics^11^, epidemiology^12^, or phylogeography^13^. The traditional approach for quantifying kinship likelihood ratios relies on establishing a value corresponding to the decreased probability for each additional repeat difference between parental and filial alleles. This so-called “mutation range” parameter is considered in diverse software^14,15^. Despite the lack of statistical support, 0.1 is sometimes suggested as an overall value for the mutation range, meaning that a two-step mutation is 10 times rarer than a single-step one, and a three-step mutation is 10 times rarer than a two-step one, and so on.

To investigate the impact of the composition of the STR’s repetitive motif in the mutational dynamics, we have compiled data available for STRs located in the non-recombining region of the Y chromosome (Y-STRs). This region of the Y chromosome possesses no homologous region on the X chromosome and, as such, they do not undergo recombination during meiosis. Hence, in simple sequence markers, any change detected between father and son must be due to a mutation event. It is also noteworthy that the data obtained for this study were generated through genotyping platforms that do not discriminate variation in sequence, but just differences in alleles’ length (automated fragment size determination after capillary electrophoresis).

Indeed, the Y chromosome is an invaluable tool for the study of germinal mutations and their biological mechanisms since it is exclusively transmitted through the paternal lineage in a haploid fashion. The NRY contains many STRs. When typing platforms discriminate solely the length of the allele, the Y chromosome, due to its specific mode of transmission, is the only component of the nuclear genome that allows the exact knowledge of which parental allele resulted in which filial one, allowing the unambiguous identification of any length mutation^16^.

In both autosomal and heterosomal modes of transmission, when no Mendelian incompatibilities are detected in parent(s)-child duos or trios, it is assumed that no mutation occurred. This unavoidably leads to an underestimation of the mutation rates, since ‘hidden’ or ‘covert’ mutations may be present^17–19^.

A most parsimonious approach is used when classifying the mutation as either single- or multistep, i.e., the mutation that requires the minimum number of steps to conciliate the observations with Mendelian transmission is assumed. This leads to an overestimation of the single-step mutation rates and a corresponding underestimation of those involving multiple steps. It is however noteworthy that this is more severe for autosomal than for X-chromosomal markers, since in father-daughter and mother-son transmissions the parental and filial alleles, respectively, are known^20^.

Here we intend to contribute to the improvement of the estimates and the mutational model design, by correlating the Y chromosome-specific STRs (Y-STRs) repetitive motif sequence, rather than just its length, with the mutational dynamics.

We have found that the frequency of multistep mutations varies widely across repeat motif compositions and length, reaching differences by a factor of nearly an order of magnitude. The implications of these findings in the fields of population genetics, epidemiology, or phylogeography, and in general evolutionary studies where STR mutation models are discussed.

## Material and methods

Data from 44 published reports^21–64^ were gathered, comprising a total of 2,444 observed mutations in 476,306 allele transfers between father and son pairs, regarding 64 Y-STRs (see Tables S1 and S2). These data were obtained in genotyping platforms (automated fragment size determination after capillary electrophoresis) that do not discriminate variation in sequence, but just size differences. As previously referred, a change between a pair of paternal and filial Y-STR alleles implies that a mutation occurred. However, a correspondence between the paternal and filial alleles only indicates the absence of mutation in simple structure STRs (harboring a single repetitive motif). For STRs with a complex structure (having two or more adjacent repetitive motifs) two mutations may occur in opposite directions, maintaining the final size of the PCR amplicon. Hence, only using STRs with simple structure is possible to determine the number of repeats involved in the allelic transmission. Thus, after compiling data from all studies including father-child duos, DYS389II, DYS390, DYS435, DYS446, DYS447, DYS520, DYS547, DYS552, and DXY156 were excluded from the analyses because they harbor complex structures. Markers containing several loci (multi-copy), such as DYS385a/b, DYS459a/b, DYS464a/b/c/d, DYS526a/b, DYS527a/b, DYF387S1, DYF399S1, DYF404S1, DYF403S1a/b, were also not considered since they do not allow the unambiguous assignment of mutation to each locus. Structure groups with fewer than 10 reported mutations were also removed from the analyses: DYS413, YCAII, DYS531 and DYS587, DYS443, DYS505 due to a lack of statistical power. Finally, DYS622, DYS630 and DYS640 were not considered since no sequence information was found.

A final subset of 35 Y-STRs, 323,818 allele transfers and 1297 mutations, was then considered for further analyses (see Table S1).

STRs were grouped according to the sequence and length of the repetitive motif present in the leading strand (retrieved from GRCh38.p14^65^), resulting in 8 groups, as shown in Table 1.

**Table 1:** Grouping of the STRs analyzed according to the repetitive motif present in the leading strand.

Group	Structure groups	Markers
ATT	AAT/ATA/TAA	DYS388
		DYS392
CTT	CTT/TCT/TTC	DYS481
		DYS612
GATA	GATA/AGAT/TAGA/ATAG	DYS393
		DYS456
		DYS522
		DYS635
		DYS439
		DYS389I
		DYS549
		DYS444
		DYS510
TCTA	TCTA/CTAT/TATC/ATCT	DYS19
		DYS391
		DYS437
		DYS533
		GATA H4
		DYS460
		DYS461
		GATA A10
		DYS434
GAAA	GAAA/AGAA/AAGA/AAAG	DYS576
		DYS627
		DYS518
		DYS458
		DYS626
		DYS722
CTTT	CTTT/TCTT/TTCT/TTTC	DYS570
		DYS449
		DYS557
		DYS709
CTTTT	CTTTT/TCTTT/TTCTT/TTTCT/TTTTC	DYS643
		DYS438
AGAGAT	AGAGAT	DYS448

In forensic genetics, STRs nomenclature recommendations state that, although most times it is possible to define different repetitive motifs within a 5’ to 3’ strand, the repeat sequence motif must be defined so that the first 5’-nucleotides that can represent a repetitive motif are used^66^. However, when a mutation occurs, it is impossible to discern if the length change resulted in the addition or deletion of the designated repetitive motif or any other. For example, if the repetitive motif of an STR is defined as TCTA, when a length mutation occurs, that repetitive motif might have been the one involved in the mutation, but so could the motifs CTAT, TATC, and ATCT (see Table 1 for the group information). It is impossible to discern which motif was involved in the mutation through capillary electrophoresis or sequencing. As such, in this work, STRs were grouped according to their structure and not their official nomenclature.

As TCTA and GATA, and GAAA and CTTT are complementary sequences, to determine if they could be grouped, Fisher exact tests were performed to ascertain the statistical significance of the differences in the number of single- and multistep mutations between the two pairs (α = 0.05). No significant differences were detected in the comparison of GAAA with CTTT markers (p = 0.8415) nor in the comparison of TCTA with GATA markers (p = 0.0846). Hence, GAAA were grouped with CTTT markers, and GATA were grouped with TCTA markers.

The ratio between single- and multistep mutations was calculated for each of the above-defined groups of markers. Fisher’s exact tests were also used to measure the significance (α = 0.05) of the single/multistep proportions between groups of markers.

The number of repeats involved in allele transitions where mutations were observed was also analyzed for the complete set of 35 single-copy Y-STRs with simple structure.

In markers DYS19, DYS389I, and DYS635, allele calling includes the total number of repeats in polymorphic and contiguous non-polymorphic tracts. Proper adjustments were made for these markers to obtain the number of repeats of the polymorphic tract.

Some of the published reports^51,56,58,61,63^ do not indicate the alleles observed in the mutation, providing only information on the type of mutation observed (single- or multistep, gain or loss of repeats). These works were thus not included in the analyses involving the number of repeats.

## Results and discussion

Although many studies report single-step mutations as much more frequent than multistep mutations, these results are usually presented as an overall value, and not analyzed per marker—see for example^23,24,38^. Our results regarding markers with simple structure show that, indeed, single-step mutations are more frequent than multistep ones (except for marker DYS438, see Table S1). However, the ratio between single- and multistep mutations varies widely between markers and groups of markers defined by their repetitive motif structure (see Table 2).

**Table 2:** Number of multistep (a) and total (b) mutations observed, multistep mutation frequency, and corresponding 95% confidence intervals, per group of markers.

Group	Multistep mutations (a)	Mutations (b)	Multistep mutation frequency*	IC 95% lower bound	IC 95% upper bound
ATT	2	17	0.1176	0.0146	0.3644
CTT	10	86	0.1162	0.0572	0.2035
TCTA/GATA	5	540	0.0093	0.0030	0.0215
GAAA/CTTT	33	629	0.0524	0.0364	0.0729
CTTTT	4	16	0.2500	0.0727	0.5238
AGAGAT	1	19	0.0526	0.0013	0.2603

*Calculated as: $\frac{a}{b}$. Values rounded to 4 dp.

The CTTTT group showed the highest frequency of multistep mutations (25% of the mutations observed), more than twice the corresponding frequency of the ATT and CTT groups, with the second-highest frequency (~ 12%). The lowest frequency of multistep mutations was observed for the group TCTA/GATA (~ 0.93%).

Comparing the two tetrameric groups, the GAAA/CTTT group showed 5.7 times higher multistep mutation frequency than the group GATA/TCTA, the corresponding confidence intervals not intersecting each other.

Ballantyne et al.^67^ concluded that motifs with strong purine:pyrimidine asymmetries have the highest diversity and variance. Our results indicate that this could also be a factor affecting the type of mutation, with a consequent impact on the variance in the number of repeats. For STRs with tetrameric motifs, the GAAA group, with a 4:0 ratio of purine:pyrimidine, presents a greater frequency of multistep mutations than the GATA group, with a 3:1 ratio (p < 0.0001, see Table 3). The same trend is observed for trimeric repeats, with the CTT having a higher ratio of multistep mutations than the ATT motifs. The frequency of multistep mutations is even higher regarding the pentameric motif CTTTT, with 0:5 ratio of purine:pyrimidine. However, in this case, we cannot discern if this difference is influenced by the higher asymmetry or the larger number of nucleotides in the motif. Significant differences were also found between both ATT and CTT groups and the TCTA/GATA (p = 0.0168 and p < 0.0001, respectively), and between both TCTA/GATA and GAAA/CTTT groups and the CTTTT (p < 0.0001, and p = 0.0102, respectively) – see Table 3.

**Table 3:** P-values resulting from a pairwise Fisher test of the number of single- and multistep mutations between the STR groups defined by the repetitive motif (α = 0.05).

	ATT	CTT	TCTA/GATA	GAAA/CTTT	CTTTT	AGAGAT
ATT	–	1.0000	**0.0168**	0.2336	0.3983	0.5929
CTT	–	–	** < 0.0001**	**0.0281**	0.2268	0.6844
TCTA/GATA	–	–	–	** < 0.0001**	** < 0.0001**	0.1881
GAAA/CTTT	–	–	–	–	**0.0102**	1.0000
CTTTT	–	–	–	–	–	0.1558
AGAGAT	–	–	–	–	–	–

Significant p-values bold, values presented with 4 dp for non-null approximate values, in which case a maximum value with one significant digit is shown.

The correlation between the length of repetitive motif and the mutation rate have been shown in different studies (e.g.^67–70^). Most of these studies also acknowledge the presence of mutations that escape SMM, however, without relating their frequency with the repetitive structure of the locus. Beyond these analyses, our work shows how frequently some STRs can escape the SMM. Most mutations obey the SMM, but some escape this model, for some markers and/or groups of markers more than for others. So, despite being the most used model, and suitable for most STRs, the SMM should be used with caution for others.

Martins et al*.*^71^ found that wild-type Machado-Joseph Disease alleles do not follow the single-step mutation model. Their results show that the frequency distribution of CAG alleles has been shaped by a multistep mutation mechanism. Indeed, this seems to be the case for some of the groups in this work, that show multistep mutation proportions up to 25%.

Most works show a considerable disproportion between single- and multistep mutations, which might be due to the high number of GATA markers analyzed in the most used multiplexes. In the last years, more GAAA markers have been added to the commercially available typing kits and the ratio between single- and multistep mutations will likely tend to be less disproportionate. Penta and hexameric motifs are much less represented in the generally used commercial kits and so their effect on these overall rates has little impact.

The number of single- and multistep mutations considering the number of repeats involved in the allele transmissions were analyzed for the complete set of markers and structure groups—see Table 4.

**Table 4:** Number of single- and multistep mutations considering the number of repeats of the parental alleles for all the structure groups.

Number of repeats	Structure group											
	CTAT/GATA		GAAA/CTTT		ATT		CTT		GAAAA		AGAGAT	
	Single	Multi	Single	Multi	Single	Multi	Single	Multi	Single	Multi	Single	Multi
6	1	0										
7	1	0										
8	3	0										
9	17	0										
10*	43	0			0	1			4	1		
11	112	3			2	0						
12**	86	0			1	0			1	3		
13	85	0			6	0						
14	49	0	1	0	2	1						
15	41	0	10	0								
16	24	0	20	1								
17	15	1	62	5								
18	3	0	69	2							2	0
19^§^	0	1	57	4							7	0
19.2			1	0								
20			26	5			1	0			3	0
21			36	1			3	0			1	1
22			23	1			2	1			1	0
23			15	0			4	1				
24			7	0			16	1				
25			3	0			7	2				
26			2	0			15	0				
27			3	1			10	3				
28			2	0			5	0				
29			10	0			0	0				
30			11	0			0	0				
31^§§^			15	0			0	1				
32			21	0								
33			20	1								
34			11	0								
35			10	1								
36			3	0								
37			8	1								
38			12	2								
39			15	0								
40			11	2								
41			10	1								
42			9	0								
43			5	0								
44			1	1								
45			0	1								

* to ** significant p-values found (α = 0.05), *p = 0.0227 for CTAT/GATA and ATT, **p < 0.0001 for CTAT/GATA and GAAAA.

^§^ to ^§§^ nearly significant p-values found (α = 0.05), ^§^p = 0.0806 for CTAT/GATA and GAAA/CTTT, ^§§^p = 0.0625 for GAAA/CTTT and CTT.

The high number of categories considered through this approach implies a low number of observations in each of them. This implies that differences may not be detected even if they exist. Nevertheless, for a set of 22 numbers of repeats existing in at least 2 structure groups, 2 showed statistically significant differences (and 2 nearly significant). This supports that, at least in some cases, the structure of the repeat motif does influence the proportion of single- and multistep mutation, beyond the length of the polymorphic tract.

## Conclusions

So far, diverse studies have shown the influence of several factors on STRs mutation rates, such as the allele length, repeat motif size and sequence, parental sex, and age. Others have studied the correlation between the mutation rate and the nucleotide composition of the repetitive motif with the same number of base pairs (see, for example,^67^). However, the influence of nucleotide composition of the repetitive motif on the type of mutation (single- or multistep), was not systematically investigated. In this study, we took advantage of the mode of transmission of the non-recombining region of the Y chromosome, which enables the direct analysis of length mutations in markers with simple structure.

Despite the inescapable problem regarding the low number of observations when modeling rare events, this work supports that, just like mutation rates, the type of mutation (single- or multistep) is heterogeneous across STRs. This includes markers with the same length of the repetitive motif, as well as alleles with the same number of repeats, although from different markers. Comparing repetitive motifs with different sizes prevent us to discern the reason leading to the observed unbalance between single- and multistep mutations. In any case, our work supports that the best fitting mutation model varies between markers.

The monomeric tract in motifs ATT, CTT, GAAA and CTTTT might be influencing slippage, or another mutation model might be operating since in these motifs the multistep mutation frequency is higher.

Most noteworthy is the case of one of the pentameric markers analyzed, DYS438, which does not fit the single-step mutation model, as half of the observed mutations involved several steps.

It is clear that, at least for some STR motif structures, the single stepwise mutation model represents, at best, a crude and biased oversimplification. The implications are manifold and affect many areas of study, such as human population and evolutionary history, genealogical studies, or forensics. Concerning forensic applications, the “mutation range” parameter of 0.1 frequently used in kinship computations seems to be too high for all tetrameric STRs analyzed and too low for pentameric ones. Based on the available data, the mutation range parameter estimates are 0.1333 for ATT markers, 0.1316 for CTT, 0.0554 for GAAA/CTTT, 0.0093 for motif TCTA/GATA, 0.3333 for CTTTT, and 0.0556 for AGAGAT (although in these two last cases more data are needed for a sound estimate).

The development of new models of STR evolution including all major factors known to influence mutation is challenging, but their development is crucial. Large datasets are needed to test mutation models and to estimate rates more accurately. One major setback is that some markers have extremely low mutation rates and gathering enough data is challenging, in such cases targeted analyses are needed. Moreover, guidelines concerning mutation reporting should be established, a need particularly felt when dealing with STRs outside NRY, as previously mentioned in^72^. These data should include parental age, and genotypic information, as the absolute frequencies of the observed alleles in one-generation profiles (separately for duos and trios in the case of either autosomal or X chromosomal markers, comprising all the cases, with or without mutation, and for the full set of analyzed markers). Such enriched and organized datasets would improve mutation modeling, enabling allele-specific mutation rates estimates, and allowing the discernment and quantification of the effects of the various factors influencing the fidelity of the genetic transmission.

## Supplementary Information

Supplementary Information 1.

Supplementary Information 2.

## Data Availability

All data generated and/or analyzed during this study are included either in the main text or Supplementary Information files, or in the main text or supplementary files of the works referenced.

## References

[1] Ellegren H (2004). Microsatellites: Simple sequences with complex evolution. Nat. Rev. Genet..

[2] Panzer S, Kuhl DPA, Caskey CT (1995). Unstable triplet repeat sequences: A source of cancer mutations?. Stem Cells.

[3] Pearson CE, Edamura KN, Cleary JD (2005). Repeat instability: Mechanisms of dynamic mutations. Nat. Rev. Genet..

[4] Schlötterer C, Tautz D (1992). Slippage synthesis of simple sequence DNA. Nucleic Acids Res..

[5] Strand M, Prolla TA, Liskay RM, Petes TD (1993). Destabilization of tracts of simple repetitive DNA in yeast by mutations affecting DNA mismatch repair. Nature.

[6] Eckert KA, Hile SE (2009). Every microsatellite is different: Intrinsic DNA features dictate mutagenesis of common microsatellites present in the human genome. Mol. Carcinog..

[7] Ohta T, Kimura M (1973). A model of mutation appropriate to estimate the number of electrophoretically detectable alleles in a finite population. Genet. Res..

[8] Wehrhahn CF (1975). The evolution of selectively similar electrophoretically detectable alleles in finite natural populations. Genetics.

[9] Gymrek M, Willems T, Reich D, Erlich Y (2017). Interpreting short tandem repeat variations in humans using mutational constraint. Nat. Genet..

[10] Sun JX (2012). A direct characterization of human mutation based on microsatellites. Nat. Genet..

[11] Xu H, Fu YX (2004). Estimating effective population size or mutation rate with microsatellites. Genetics.

[12] Aandahl RZ, Reyes JF, Sisson SA, Tanaka MM (2012). A model-based bayesian estimation of the rate of evolution of VNTR loci in mycobacterium tuberculosis. PLoS Comput. Biol..

[13] Barthe S, Gugerli F, Barkley NA, Maggia L, Cardi C, Scotti I (2012). Always look on both sides: Phylogenetic information conveyed by simple sequence repeat allele sequences. PLoS ONE.

[14] “Forensic Mathematics.” https://dna-view.com/#Software (Accessed Apr 28, 2022).

[15] Kling D, Tillmar AO, Egeland T (2014). Familias 3: Extensions and new functionality. Forensic Sci. Int. Genet..

[16] Pinto N, Gusmão L, Amorim A (2014). Mutation and mutation rates at y chromosome specific Short Tandem Repeat Polymorphisms (STRs): A reappraisal. Forensic Sci. Int. Genet..

[17] Chakraborty R, Stivers DN, Zhong Y (1996). Estimation of mutation rates from parentage exclusion data: Applications to STR and VNTR loci. Mutat. Res..

[18] Vicard P, Dawid AP (2004). A statistical treatment of biases affecting the estimation of mutation rates. Mutat. Res..

[19] Brenner CH (2004). Multiple mutations, covert mutations and false exclusions in paternity casework. Int. Congr. Ser..

[20] Antão-Sousa S, Conde-Sousa E, Gusmão L, Amorim A, Pinto N (2019). Underestimation and misclassification of mutations at X chromosome STRs depend on population’s allelic profile. Forensic Sci. Int. Genet. Suppl. Ser..

[21] Lessig R, Edelmann J (1998). Y chromosome polymorphisms and haplotypes in west Saxony (Germany). Int. J. Leg. Med..

[22] Blanchi NO (1998). Characterization of ancestral and derived Y-chromosome haplotypes of new world native populations. Am. J. Hum. Genet..

[23] Kayser M (2000). Characteristics and frequency of germline mutations at microsatellite loci from the human Y chromosome, as revealed by direct observation in father/son pairs. Am. J. Hum. Genet..

[24] Dupuy BM, Stenersen M, Egeland T, Olaisen B (2004). Y-chromosomal microsatellite mutation rates: Differences in mutation rate between and within loci. Hum. Mutat..

[25] Tsai LC (2002). Haplotype frequencies of nine Y-chromosome STR loci in the Taiwanese Han population. Int. J. Leg. Med..

[26] Kurihara R (2004). Mutations in 14 Y-STR loci among Japanese father-son haplotypes. Int. J. Leg. Med..

[27] Budowle B (2005). Twelve short tandem repeat loci Y chromosome haplotypes: Genetic analysis on populations residing in North America. Forensic Sci. Int..

[28] Berger B, Lindinger A, Niederstätter H, Grubwieser P, Parson W (2005). Y-STR typing of an Austrian population sample using a 17-loci multiplex PCR assay. Int. J. Leg. Med..

[29] Gusmão L (2005). Mutation rates at Y chromosome specific microsatellites. Hum. Mutat..

[30] Ballard DJ (2005). A study of mutation rates and the characterisation of intermediate, null and duplicated alleles for 13 Y chromosome STRs. Forensic Sci. Int..

[31] Turrina S, Atzei R, de Leo D (2006). Y-chromosomal STR haplotypes in a Northeast Italian population sample using 17plex loci PCR assay. Int. J. Leg. Med..

[32] Lee HY (2007). Haplotypes and mutation analysis of 22 Y-chromosomal STRs in Korean father-son pairs. Int. J. Leg. Med..

[33] Hohoff C, Dewa K, Sibbing U, Hoppe K, Forster P, Brinkmann B (2007). Y-chromosomal microsatellite mutation rates in a population sample from northwestern Germany. Int. J. Leg. Med..

[34] Kumagai R, Kumagai A, Saigusa K, Aoki Y (2007). Haplotype analysis of 17 Y-STR loci in a Japanese population. Forensic Sci. Int..

[35] Pontes ML, Cainé L, Abrantes D, Lima G, Pinheiro MF (2007). Allele frequencies and population data for 17 Y-STR loci (AmpFlSTR Y-filer) in a Northern Portuguese population sample. Forensic Sci. Int..

[36] Sen Shi M, Tang JP, Bai RF, Yu XJ, Lv JY, Hu B (2007). Haplotypes of 20 Y-chromosomal STRs in a population sample from southeast China (Chaoshan area). Int. J. Leg. Med..

[37] Domingues PM, Gusmão L, da Silva DA, Amorim A, Pereira RW, de Carvalho EF (2007). Sub-Saharan Africa descendents in Rio de Janeiro (Brazil): Population and mutational data for 12 Y-STR loci. Int. J. Leg. Med..

[38] Decker AE, Kline MC, Redman JW, Reid TM, Butler JM (2008). Analysis of mutations in father-son pairs with 17 Y-STR loci. Forensic Sci. Int. Genet..

[39] Sánchez-Diz P (2008). Population and segregation data on 17 Y-STRs: Results of a GEP-ISFG collaborative study. Int. J. Leg. Med..

[40] Padilla-Gutiérrez JR (2008). Population data and mutation rate of nine Y-STRs in a mestizo Mexican population from Guadalajara, Jalisco, México. Leg. Med..

[41] Goedbloed M (2009). Comprehensive mutation analysis of 17 Y-chromosomal short tandem repeat polymorphisms included in the AmpFlSTR Yfiler PCR amplification kit. Int. J. Leg. Med..

[42] Ge J, Budowle B, Aranda XG, Planz JV, Eisenberg AJ, Chakraborty R (2009). Mutation rates at Y chromosome short tandem repeats in Texas populations. Forensic Sci. Int. Genet..

[43] Kim SH (2009). Population genetics and mutational events at 6 Y-STRs in Korean population. Forensic Sci. Int. Genet..

[44] Ballantyne KN (2014). Toward male individualization with rapidly mutating y-chromosomal short tandem repeats. Hum. Mutat..

[45] Robino C (2015). Development of an Italian RM Y-STR haplotype database: Results of the 2013 GEFI collaborative exercise. Forensic Sci. Int. Genet..

[46] Wang Y (2016). Genetic polymorphisms and mutation rates of 27 Y-chromosomal STRs in a Han population from Guangdong Province, Southern China. Forensic Sci. Int. Genet..

[47] Antão-Sousa S (2017). Mutation rates and segregation data on 16 Y-STRs: An update to previous GHEP-ISFG studies. Forensic Sci. Int. Genet. Suppl. Ser..

[48] Adnan A, Rakha A, Lao O, Kayser M (2018). Mutation analysis at 17 Y-STR loci (Yfiler) in father-son pairs of male pedigrees from Pakistan. Forensic Sci. Int. Genet..

[49] Bugoye FC, Mulima E, Misinzo G (2018). Analysis of mutation rate of 17 Y-chromosome short tandem repeats loci using Tanzanian father-son paired samples. Genet. Res. Int..

[50] Mertoglu E, Filoglu G, Zorlu T, Bulbul O (2018). Estimation of the Y-chromosomal short tandem repeat (Y-STR) mutation rates in Turkey. Turk. J. Biochem..

[51] Yang Y (2018). Haplotypic polymorphisms and mutation rate estimates of 22 Y-chromosome STRs in the Northern Chinese Han father–son pairs. Sci. Rep..

[52] Wu W (2018). Mutation rates at 42 Y chromosomal short tandem repeats in Chinese Han population in Eastern China. Int. J. Leg. Med..

[53] Petrovic V, Kecmanovic M, Keckarevic Markovic M, Keckarevic D (2019). Assessment of mutation rates for PPY23 Y chromosome STR loci in Serbian father-son pairs. Forensic Sci. Int. Genet..

[54] Zhang J (2019). Mutation rates in father-son pairs of the 27 Y-STR loci in the Dezhou Han population from Shandong province, eastern China. J. Forensic Leg. Med..

[55] Ay M, Serin A, Sevay H, Gurkan C, Canan H (2019). Genetic characterisation of 13 rapidly mutating Y-STR loci in 100 father and son pairs from South and East Turkey. Ann. Hum. Biol..

[56] Yuan L (2019). Mutation analysis of 13 RM Y-STR loci in Han population from Beijing of China. Int. J. Leg. Med..

[57] Ambrosio IB (2020). Mutational data and population profiling of 23 Y-STRs in three Brazilian populations. Forensic Sci. Int. Genet..

[58] Lin H, Ye Q, Tang P, Mo T, Yu X, Tang J (2020). Analyzing genetic polymorphism and mutation of 44 Y-STRs in a Chinese Han population of Southern China. Leg. Med..

[59] Fu J, Cheng J, Wei C, Khan MA, Jin Z, Fu J (2020). Assessing 23 Y-STR loci mutation rates in Chinese Han father-son pairs from southwestern China. Mol. Biol. Rep..

[60] Bredemeyer S, Roewer L, Willuweit S (2021). Next generation sequencing of Y-STRs in father-son pairs and comparison with traditional capillary electrophoresis. Forensic Sci. Res..

[61] Vieira-Silva C, Dario P, Ribeiro T, Lucas I, Geada H, Espinheira R (2009). Y-STR mutational rates determination in South Portugal Caucasian population. Forensic Sci. Int. Genet. Suppl. Ser..

[62] Soares-Vieira JA, Billerbeck AEC, Iwamura ESM, Mendonca BB, Gusmão L, Otto PA (2008). Population and mutation analysis of Y-STR loci in a sample from the city of São Paulo (Brazil). Genet Mol Biol.

[63] Laouina A (2013). Mutation rate at 17 Y-STR loci in ‘Father/Son’ pairs from moroccan population. Leg. Med..

[64] Dupuy BM (2001). Y-chromosome variation in a Norwegian population sample. Forensic Sci. Int..

[65] “GRCh38.p14 - hg38 - Genome - Assembly - NCBI.” https://www.ncbi.nlm.nih.gov/assembly/GCF_000001405.40 (Accessed Apr 25, 2022).

[66] Bar W (1997). DNA recommendations. Further report of the DNA Commission of the ISFH regarding the use of short tandem repeat systems. International Society for Forensic Haemogenetics. Int. J. Legal Med..

[67] Ballantyne KN (2010). Mutability of Y-chromosomal microsatellites: Rates, characteristics, molecular bases, and forensic implications. Am. J. Hum. Genet..

[68] Chakraborty R, Kimmel M, Stivers DN, Davison LJ, Deka R (1997). Relative mutation rates at di-, tri-, and tetranucleotide microsatellite loci. Proc. Natl. Acad. Sci. USA.

[69] Pemberton TJ, Sandefur CI, Jakobsson M, Rosenberg NA (2009). Sequence determinants of human microsatellite variability. BMC Genom..

[70] Webster MT, Smith NGC, Ellegren H (2002). Microsatellite evolution inferred from human-chimpanzee genomic sequence alignments. Proc. Natl. Acad. Sci. USA.

[71] Martins S, Calafell F, Wong VC, Sequeiros J, Nio Amorim A (2006). A multistep mutation mechanism drives the evolution of the CAG repeat at MJD/SCA3 locus. Eur. J. Hum. Genet..

[72] Amorim A, Pinto N (2020). Estimates of mutation rates from incompatibilities are misleading - guidelines for publication and retrieval of mutation data urgently needed. Forensic Sci. Int..

