# Supplementary material for: The sequence of the repetitive motif influences the frequency of multistep mutations in Short Tandem Repeats
Source: Sci Rep. 2023 Jun 24;13:10251. doi: 10.1038/s41598-023-32137-y (PMC10290632; doi:10.1038/s41598-023-32137-y)
Supplement: Supplementary file 1 — Supplementary Information 1. [file 41598_2023_32137_MOESM1_ESM.docx]

**Table S1:** Number of mutations and allelic transmissions gathered from the 44 published works and the mutational steps for each mutation.

| Structure group | Markers | Number of mutations observed | Number of allelic transmissions | Number of mutational steps | | | | | | |
| --- | --- | --- | --- | --- | --- | --- | --- | --- | --- | --- |
|  |  |  |  | **1 step** | **2 steps** | **3 steps** | **4 steps** | **5 steps** | **6 steps** | **NC*** |
| CA | DYS413 (YCAIII)^§^ | 1 | 100 | 0 | 1 | 0 | 0 | 0 | 0 | 0 |
|  | YCAII^§^ | 2 | 2,296 | 2 | 0 | 0 | 0 | 0 | 0 | 0 |
| ATT | **DYS392** | 14 | 22,044 | 13 | 0 | 0 | 1 | 0 | 0 | 0 |
|  | **DYS388** | 3 | 3,713 | 2 | 1 | 0 | 0 | 0 | 0 | 0 |
| CTT | **DYS481** | 18 | 3,901 | 15 | 3 | 0 | 0 | 0 | 0 | 0 |
|  | **DYS612** | 68 | 4,056 | 61 | 7 | 0 | 0 | 0 | 0 | 0 |
| GATA | **DYS19** | 53 | 24,148 | 52 | 0 | 0 | 0 | 0 | 0 | 1 |
|  | **DYS389I** | 61 | 23,929 | 61 | 0 | 0 | 0 | 0 | 0 | 0 |
|  | DYS390^§^ | 57 | 22,879 | 56 | 1 | 0 | 0 | 0 | 0 | 0 |
|  | **DYS391** | 62 | 23,567 | 60 | 2 | 0 | 0 | 0 | 0 | 0 |
|  | **DYS393** | 29 | 22,113 | 29 | 0 | 0 | 0 | 0 | 0 | 0 |
|  | **DYS437** | 21 | 18,793 | 21 | 0 | 0 | 0 | 0 | 0 | 0 |
|  | **DYS439** | 94 | 18,887 | 94 | 0 | 0 | 0 | 0 | 0 | 0 |
|  | **DYS456** | 66^A^ | 15,667 | 64 | 0 | 1 | 0 | 0 | 0 | 0 |
|  | **DYS460** | 19 | 4,297 | 19 | 0 | 0 | 0 | 0 | 0 | 0 |
|  | **DYS461** | 0 | 873 | 0 | 0 | 0 | 0 | 0 | 0 | 0 |
|  | **DYS533** | 8 | 4,863 | 7 | 1 | 0 | 0 | 0 | 0 | 0 |
|  | **DYS549** | 7 | 1,811 | 7 | 0 | 0 | 0 | 0 | 0 | 0 |
|  | **DYS635 (C4)** | 51 | 16,643 | 51 | 0 | 0 | 0 | 0 | 0 | 0 |
|  | **GATA A10** | 4 | 1,026 | 4 | 0 | 0 | 0 | 0 | 0 | 0 |
|  | **GATA H4** | 41^A^ | 15,388 | 39 | 1 | 0 | 0 | 0 | 0 | 0 |
|  | **DYS444** | 8 | 2,458 | 8 | 0 | 0 | 0 | 0 | 0 | 0 |
|  | **DYS522** | 4 | 1,954 | 4 | 0 | 0 | 0 | 0 | 0 | 0 |
|  | **DYS434** | 0 | 80 | 0 | 0 | 0 | 0 | 0 | 0 | 0 |
|  | **DYS510** | 6 | 1,160 | 6 | 0 | 0 | 0 | 0 | 0 | 0 |
|  | DYS552^§^ | 9 | 1,160 | 9 | 0 | 0 | 0 | 0 | 0 | 0 |
| GAAA | **DYS449** | 87 | 7,175 | 86 | 0 | 1 | 0 | 0 | 0 | 0 |
|  | **DYS458** | 110 | 15,695 | 107 | 2 | 1 | 0 | 0 | 0 | 0 |
|  | **DYS518** | 97 | 6,492 | 87 | 4 | 3 | 2 | 0 | 0 | 1 |
|  | **DYS570** | 74 | 7,862 | 66 | 6 | 1 | 0 | 0 | 0 | 1 |
|  | **DYS576** | 106 | 7,763 | 101 | 2 | 2 | 0 | 0 | 0 | 1 |
|  | **DYS626** | 41 | 4,441 | 39 | 1 | 0 | 0 | 0 | 1 | 0 |
|  | **DYS627** | 111 | 6,723 | 105 | 5 | 1 | 0 | 0 | 0 | 0 |
|  | DYS385^§^ | 107 | 42,217 | 100 | 6 | 1 | 0 | 0 | 0 | 0 |
|  | **DYS557** | 4 | 1,239 | 4 | 0 | 0 | 0 | 0 | 0 | 0 |
|  | **DYS709** | 0 | 80 | 0 | 0 | 0 | 0 | 0 | 0 | 0 |
|  | **DYS722** | 1 | 640 | 1 | 0 | 0 | 0 | 0 | 0 | 0 |
|  | DYF387S1^§^ | 66 | 12,590 | 59 | 5 | 1 | 1 | 0 | 0 | 0 |
|  | DYF399S1^§^ | 281 | 4,053 | 268 | 7 | 3 | 0 | 0 | 0 | 3 |
|  | DYF404S1^§^ | 61^B^ | 4,052 | 59 | 1 | 0 | 0 | 0 | 0 | 0 |
| GGAA | DYS464a/b/c/d^§^ | 5^B^ | 473 | 4 | 0 | 0 | 0 | 0 | 0 | 0 |
|  | DYS443^§^ | 2 | 1,240 | 2 | 0 | 0 | 0 | 0 | 0 | 0 |
|  | DYS505^§^ | 0 | 64 | 0 | 0 | 0 | 0 | 0 | 0 | 0 |
|  | DYS526a^§^ | 15 | 4,425 | 11 | 4 | 0 | 0 | 0 | 0 | 0 |
| TGGA | DYS435^§^ | 0 | 161 | 0 | 0 | 0 | 0 | 0 | 0 | 0 |
| TAAA | DYS459a/b^§^ | 1 | 2,323 | 1 | 0 | 0 | 0 | 0 | 0 | 0 |
|  | DYS531^§^ | 0 | 80 | 0 | 0 | 0 | 0 | 0 | 0 | 0 |
| GAAAA | **DYS438** | 9 | 18,808 | 4 | 3 | 0 | 1 | 0 | 0 | 1 |
|  | **DYS643** | 1 | 1,172 | 1 | 0 | 0 | 0 | 0 | 0 | 0 |
| TAAAA | DXY156^§^ | 0 | 1,027 | 0 | 0 | 0 | 0 | 0 | 0 | 0 |
| AGAGA | DYS446^§^ | 7 | 1,817 | 7 | 0 | 0 | 0 | 0 | 0 | 0 |
| ATACA | DYS587^§^ | 1 | 1,160 | 1 | 0 | 0 | 0 | 0 | 0 | 0 |
| AGAGAT | **DYS448** | 19 | 14,357 | 18 | 1 | 0 | 0 | 0 | 0 | 0 |
| Complex structure combining different motifs ^§^ | DYS447^§^ | 4 | 2,394 | 4 | 0 | 0 | 0 | 0 | 0 | 0 |
|  | DYS389II^§^ | 83 | 23,898 | 80 | 2 | 1 | 0 | 0 | 0 | 0 |
|  | DYS520^§^ | 2 | 1,241 | 2 | 0 | 0 | 0 | 0 | 0 | 0 |
|  | DYS526b^§^ | 50 | 4,401 | 49 | 1 | 0 | 0 | 0 | 0 | 0 |
|  | DYS527^§^ | 18 | 3,494 | 16 | 2 | 0 | 0 | 0 | 0 | 0 |
|  | DYF403S1a^§^ | 120 | 3,889 | 111 | 8 | 1 | 0 | 0 | 0 | 0 |
|  | DYF403S1b^§^ | 36^C^ | 3,881 | 30 | 3 | 1 | 1 | 0 | 0 | 0 |
|  | DYS547^§^ | 69^B^ | 4,053 | 67 | 0 | 0 | 1 | 0 | 0 | 0 |
|  | DYS622^§^ | 3 | 1,240 | 3 | 0 | 0 | 0 | 0 | 0 | 0 |
|  | DYS630^§^ | 10 | 1,240 | 10 | 0 | 0 | 0 | 0 | 0 | 0 |
|  | DYS640^§^ | 0 | 640 | 0 | 0 | 0 | 0 | 0 | 0 | 0 |

NC* – Non-consensual number of repeats mutation, involving non-integer number of repeats.

^A^ – One mutation without allelic information

^B^ – Indeterminate/unspecified mutation

^C^ – One mutation is a locus deletion in the son

^§^ - Not further considered in the analyses
